# Transcriptomic and Single-Cell Analysis Reveals Regulatory Networks and Cellular Heterogeneity in Mouse Primary Sjögren’s Syndrome Salivary Glands

**DOI:** 10.3389/fimmu.2021.729040

**Published:** 2021-11-29

**Authors:** Erich Horeth, Akinsola Oyelakin, Eun-Ah Christine Song, Monika Che, Jonathan Bard, Sangwon Min, Jeremy Kiripolsky, Jill M. Kramer, Satrajit Sinha, Rose-Anne Romano

**Affiliations:** ^1^ Department of Oral Biology, State University of New York at Buffalo, Buffalo, NY, United States; ^2^ Genomics and Bioinformatics Core, State University of New York at Buffalo, Buffalo, NY, United States; ^3^ Department of Biochemistry, State University of New York at Buffalo, Buffalo, NY, United States

**Keywords:** primary Sjögren’s syndrome, salivary glands, RNA-sequencing, single cell RNA-sequencing, gene expression, genomics

## Abstract

Sjögren’s Syndrome (SS) is a chronic autoimmune disease of unknown etiology which primarily affects the salivary and lacrimal glands resulting in the loss of secretory function. Treatment options for SS have been hampered due to the lack of a better understanding of the underlying gene regulatory circuitry and the interplay between the myriad pathological cellular states that contribute to salivary gland dysfunction. To better elucidate the molecular nature of SS, we have performed RNA-sequencing analysis of the submandibular glands (SMG) of a well-established primary Sjögren’s Syndrome (pSS) mouse model. Our comprehensive examination of global gene expression and comparative analyses with additional SS mouse models and human datasets, have identified a number of important pathways and regulatory networks that are relevant in SS pathobiology. To complement these studies, we have performed single-cell RNA sequencing to examine and identify the molecular and cellular heterogeneity of the diseased cell populations of the mouse SMG. Interrogation of the single-cell transcriptomes has shed light on the diversity of immune cells that are dysregulated in SS and importantly, revealed an activated state of the salivary gland epithelial cells that contribute to the global immune mediated responses. Overall, our broad studies have not only revealed key pathways, mediators and new biomarkers, but have also uncovered the complex nature of the cellular populations in the SMG that are likely to drive the progression of SS. These newly discovered insights into the underlying molecular mechanisms and cellular states of SS will better inform targeted therapeutic discoveries.

## Introduction

Sjögren’s Syndrome (SS) is a chronic inflammatory autoimmune disease that commonly affects middle-aged women with a female to male ratio of up to 20:1 (1, 2). Although the etiology of SS remains largely unknown, hallmark features of this disease include lymphocytic infiltration of the salivary glands (SG) and lacrimal glands (LG), as well as loss of saliva and tear production. While this disease primarily involves the SG and LG, it can affect a wide range of other organs including the skin, kidneys, lungs and nervous system (3). Sjögren’s Syndrome can exist alone, referred to as primary Sjögren’s Syndrome (pSS), or as secondary Sjögren’s Syndrome, where it occurs in conjunction with other autoimmune connective tissue diseases (4). While SS overwhelmingly manifests as ocular and oral dryness, fatigue, and pain, patients are at elevated risk for the development of comorbidities such as pulmonary fibrosis, cardiovascular disease, B cell lymphoma, and other malignancies (5, 6).

Despite the well-characterized clinical manifestations associated with this disease, the underlying pathogenesis of pSS remains largely unknown. This is in part, due to the fact that pSS is a complex multifactorial disease that is triggered by genetic and environmental factors. Indeed, the search for genetic risk factors for pSS have led to the discovery of several alleles within the human leukocyte antigen (HLA) Class II and the major histocompatibility complex (MHC) Class II loci that are associated with pSS (7, 8). Furthermore, genome wide association studies (GWAS) have also identified numerous susceptibility genes including *STAT4*, *IRF5*, and *SH2D2A* (9). Interestingly, several of these genes have been implicated in the Interferon (IFN) signaling pathway, an important regulatory pathway previously shown to be a central player in pSS disease pathogenesis (10–12). In addition to IFN signaling, recent studies have also pointed to a prominent role for various signaling molecules including cytokines and chemokines, in directing the immune related effects associated with this disease. Notably, several chemokines including CCL19, CCL21, and CXCL13 have been shown to play key roles not only in recruiting various types of immune cells including B cells and T cells to the SGs but also in mediating their cellular responses (13–15). Despite some progress in our understanding of the molecular underpinnings of the disease, many facets of SS biology remain unexplored. This is an area of significant unmet need since current treatment options are mainly limited to symptomatic relief, and no effective cure has been developed to date (3).

Mouse models have served valuable roles in deciphering various facets of SG biology. Indeed, the last several decades have witnessed the generation of a large number of mouse models to study various aspects of human diseases affecting the SG, including pSS (16–26). Among such mouse strains, the nonobese diabetic (NOD) derived strain of mice remains perhaps one of the most extensively characterized and well-studied mouse models to investigate the pathogenesis of SS. Although the initial NOD inbred (NOD/ShiLt) strain displayed symptoms resembling SS including the presence of inflammatory cell infiltrates and impaired salivary and lacrimal secretion, these animals also developed type I diabetes (T1D) (19, 21). To circumvent the difficulties of studying SS in the background of T1D (27), we utilized a mouse model in which the NOD/ShiLt major histocompatibility complex was replaced with that of a healthy C57BL/10 strain, resulting in a congenic strain of mice (NOD.B10Sn-*H2^b^*/J or NOD.B10) that develop pSS but are protected from T1D (16). Indeed, these animals share many clinical features associated with pSS including a female disease predilection, focal lymphocytic infiltration of the SMG and lacrimal glands, reduced salivary flow and systemic disease manifestations affecting the kidney and lung (17, 28–30). Mechanistic studies performed in these mice have revealed roles for Toll Like Receptor (TLR) and Myd88 signaling pathways in promoting disease development (30–32). While these studies have informed on specific pathways and mediators of disease development, comprehensive studies aimed at uncovering global alterations in gene expression have been lacking.

To obtain a better understanding of the underlying mechanisms contributing to pSS disease development, here we have performed bulk RNA-sequencing (RNA-seq) to examine the global gene expression profiles of mouse salivary glands from control and NOD.B10 female mice. Functional gene enrichment and regulatory pathway analysis of the salivary glands revealed a number of molecular players and networks that are relevant in SS pathobiology including various cytokine and toll like receptor signaling pathways. In parallel, we have performed single-cell RNA-sequencing (scRNA-seq) to explore the cellular transcriptomic landscapes of control and NOD.B10 salivary glands. Interrogation of the single-cell transcriptomes have not only revealed the degree of cellular heterogeneity of SS glands, but have offered a glimpse into the vast number of immune cells present in diseased glands and the diverse nature of these immune cell types. Moreover, our comparative analysis of the bulk and scRNA-seq datasets has shed light on the altered gene expression profile of specific epithelial populations such as the acinar and ductal cells of the affected salivary glands. Another revealing finding from these analyses is the expression of various genes in the epithelial cells that are typically associated with innate and acquired immune responses. This observation reaffirms the prevailing notion that the activated and inflamed state of the epithelial cells in pSS is likely to play a prominent role and be a major contributing factor to disease pathophysiology (33–36). Overall, our comprehensive studies have highlighted the importance of key signaling networks and pathways and offered new insights into the underlying molecular nature of the diverse and afflicted cellular subpopulations in pSS.

## Material and Methods

### Mouse Models

All animal experiments and procedures were performed in accordance with the State University of New York at Buffalo (University at Buffalo) Institutional Animal Care and Use Committee (IACUC) regulations. All procedures were approved by University at Buffalo IACUC. NOD.B10 and C57BL/10SnJ (BL/10) control mice were purchased from Jackson Laboratories (stock numbers #002591 and 000666, respectively). Female mice were used in all experiments.

### Primary Sjögren’s Syndrome Mouse Models Examined in This Study

Both mouse models (NOD.B10 and the C57BL/6.NOD-*Aec1Aec2*) develop primary SS (pSS) and are derived from the nonobese diabetic (NOD/Lt) inbred strain, however, these animals do not share a common genetic background. Briefly, the NOD.B10 mice were generated by replacing the major histocompatibility complex (MHC) I-*A^g^* ^7^ molecule of NOD/Lt mice with MHC I-A^b^ from the C57BL/10SnJ strain, and thus these animals are congenic with the NOD/Lt mouse strain (16, 17). The C57BL/6.NOD-*Aec1Aec2* NOD-derived mouse strain were bred on a C57BL/6 background but carry two autoimmune exocrinopathy loci from the NOD/Lt mice (18).

### RNA Isolation and Quantitative RT-PCR

Total RNA was extracted from whole mouse submandibular salivary gland tissues from control BL/10 (7 months of age) and NOD.B10 (7-8 months of age) female mice as previously described (37). For quantitative reverse-transcription polymerase chain reaction (qRT-PCR) a total of 1 microgram of RNA was reverse transcribed using the iScript cDNA Synthesis Kit (Bio-Rad, 1708890) according to the manufacturer’s instructions. Quantitative reverse-transcription polymerase chain reaction was performed on a CFX96 Touch™ Real-Time PCR Detection System (Bio-Rad, 1855195) using iQ SYBR Green Supermix (Bio-Rad, 1708882). All qRT-PCR assays were performed in triplicates in at least three independent biological replicates. Relative expression values of each target gene were normalized to hypoxanthine guanine phosphoribosyltransferase (Hprt) expression. Primer sequences are provided in **Supplementary Table S22**.

### Bulk RNA-Sequencing, Differentially Expressed Gene (DEG) and Enrichment Analyses

Total RNA was extracted from whole mouse salivary gland tissues as previously described (37). For each RNA sample, 50bp cDNA-libraries were generated using the TrueSeq RNA Sample Preparation Kit (Illumina). Libraries were sequenced on the Illumina HiSeq 2500. Quality metrics were generated using FASTQC (38) v0.4.3, and high quality reads were mapped to the *Mus musculus* genome (*mm9* build) with the Tophat2 (39) v2.0.13 wrapper for Bowtie2 (40) v2.2.6. The reads that aligned to the mouse genome were counted using featureCounts (41). All RNA-seq analysis was performed as previously described (37, 42). For determining differentially expressed genes, an adjusted p-value < 0.05 based on Benjamini-Hochberg method was used as a cut-off using DESeq2. Top ranked upregulated and downregulated genes (based on fold change) were used for subsequent pathway analyses as indicated. Datasets have been deposited in the Gene Expression Omnibus (GEO) database under the accession number GEO: GSE175649.

### Single-Cell RNA Sequencing Analysis

Single cell suspensions from 1 freshly isolated female control and 1 female NOD.B10 SMGs were generated for scRNA-seq analysis as previously described (43–45). A total of 21,386 cells from control SMGs were sequenced to a depth of 339 million reads, 15,891 reads per cell and 867 median genes per cell. A total of 13,846 NOD.B10 SMG cells were sequenced to a depth of 438 million reads, 31,651 reads per cell and 456 median genes per cell. The output from 10X Genomics Cellranger version 3.0.1 pipeline was used as input into the R analysis package Seurat version 4.0.1 (46). Cells with high unique molecular index counts (nCount_RNA > 40,000), and outlier gene detection rates (nFeature_RNA 200 and > 5,000), high mitochondrial transcript load (>50%), and high transcript counts for red blood cell markers were filtered from the analysis. After filtering and down-sampling to control for variable cell capture efficiencies on the 10X platform, a total of 12,000 control cells and 10,641 NOD.B10 cells were analyzed. The data was normalized using Seurat’s logNormalize with a scale factor of 10,000. Principle component analysis (PCA) and Uniform Manifold Approximation and Projection (UMAP) algorithm was used for dimensionality reduction and visualization, followed by the construction of a Shared Nearest Neighbor (SNN) graph and clustering analysis. Using the called clusters, cluster-to-cluster differential expression testing using the Wilcoxon Rank Sum identified unique gene markers for each cluster. To validate our annotation, our control scRNA-seq dataset was mapped to additional scRNA-seq datasets that were recently reported in the mouse SMG (47). Label transfer from the Hauser et al. adult murine data set was performed by utilizing the Azimuth workflow for mapping query datasets in Seurat 4.0 release (48). Briefly, the Seurat R data object generated by Hauser et al. was used as a reference data set, leveraging the cell cluster annotations as define by Hauser et al. (47). The Hauser et al. P30 adult female cell data set was then subset from the R data object and subsequently integrated with our single-cell data set using the MapQuery function that identifies transfer anchors (or pairs of cells which are mutual nearest neighbors) which allows for sample-to-sample integration into a shared uniform manifold approximation and projection (UMAP) plot. To confirm successful label transfer, feature plots for individual marker genes were compared to confirm common expression patterns between data sets. A complete list of genes enriched per cluster using a 0.25 log2 fold change cut-off is provided in **Supplementary Tables S9, S10**. Pathway analyses was performed using DAVID and Ingenuity Pathway Analysis (IPA, QIAGEN Inc., https://www.qiagenbioinformatics.com/products/ingenuitypathway-analysis). Additionally, the lists of DEGs generated from subsets of the scRNA-seq dataset were overlayed with the DEGs identified in our bulk RNA-seq dataset in order to identify concordant DEGs (**Supplementary Tables S18–S20**). Datasets have been deposited in the Gene Expression Omnibus (GEO) database under the accession number GEO: GSE175649.

## Results

### Defining the Transcriptome of Mouse Primary Sjögren’s Syndrome Submandibular Glands

**Figure 1:**
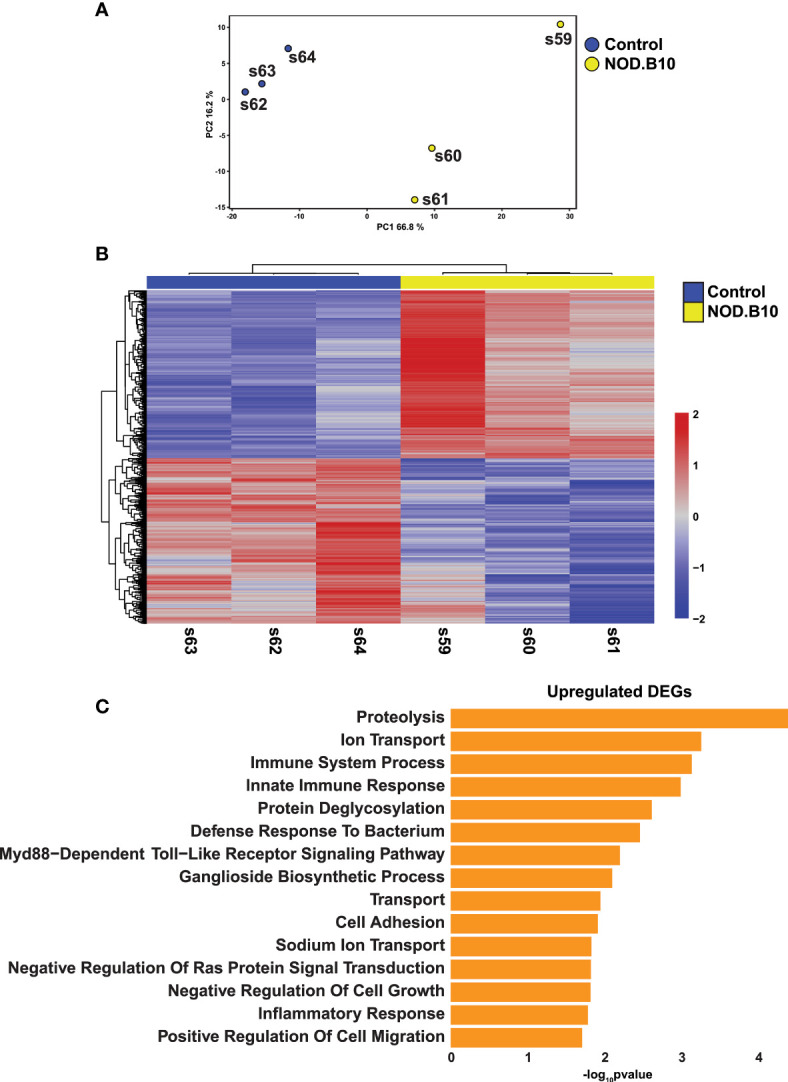
Comprehensive Transcriptomic Analysis of Submandibular Salivary Glands of NOD.B10 pSS Mice. **(A)** Plot shows Principal Component Analysis (PCA) coordinates for three control and three NOD.B10 mouse salivary glands. Blue and yellow circles represent control and NOD.B10 mice, respectively. **(B)** Heatmap visualization of differentially expressed genes (DEGs) in submandibular salivary glands of the control and NOD.B10 mice. **(C)** Bar plot highlighting enriched biological processes in the top 200 upregulated DEGs identified in panel **(B)** above.

To better define global alterations in the gene expression patterns of pSS and identify molecular players that may contribute to disease pathogenesis, we performed RNA-sequencing based profiling of 7-month-old submandibular salivary glands (SMG) from C57BL/10 (control) and NOD.B10 female mice. NOD.B10 animals have been shown to develop clinical disease by 6 months of age. Moreover, these mice acquire several associated histopathological features including focal lymphocytic infiltration of the SMG and reduced salivary flow, which is common to human pSS (17, 28, 49). We utilized three biological replicates of SMG from the control and NOD.B10 mice to capture source variability and to ensure robust downstream inferential analysis. Importantly, we also performed histological analysis of the SMGs which showed focal lymphocytic infiltration in NOD.B10 salivary glands, but not in control glands, as visualized by hematoxylin and eosin (H&E) staining (**Supplementary Figure S1**). To better analyze the RNA-seq based gene expression patterns between the control and NOD.B10 SMGs, we first utilized principal component analysis (PCA). As expected, we observed a clear separation between the two samples, highlighting the inherent differences in gene expression between the control and NOD.B10 glands (**Figure 1A**).

The generation of bulk RNA-seq data from NOD.B10 SMGs allowed us to investigate not only the altered gene expression pattern in this specific mouse model, but also examine the degree of overlap with other pSS mouse models and human pSS (described in the experimental scheme in **Supplementary Figure S2**). Towards this end, we first compared the transcriptomic profiles of the control and NOD.B10 SMGs. This analysis identified 1076 differentially expressed genes (DEGs) between the control and NOD.B10 SMGs, with 542 genes being upregulated and 534 showing downregulation at a false discovery rate (FDR) of 0.05 (**Figure 1B** and **Supplementary Table S1**). To probe the biological relevance of the transcriptomic differences between the control and NOD.B10 SMGs, we analyzed the top 200 upregulated genes, based on fold change, using the Database for Annotation, Visualization and Integrated Discovery (DAVID) (50). As expected, in the NOD.B10 SMGs we observed specific enrichment of biological processes associated with immune responses including immune system process, innate immune response, defense response to bacterium, and inflammatory response - many of which have been implicated in SS pathogenesis (51, 52) (**Figure 1C** and **Supplementary Table S2**). Of note was the observed upregulation of the Myd88-dependent toll-like receptor signaling pathway which is in good agreement with recent studies that demonstrated amelioration of local and systemic SS disease manifestations upon the loss of Myd88 expression in the NOD.B10 mouse model (30–32). In contrast, the top 200 downregulated genes based on fold change were associated with positive regulation of T cell mediated immune response to tumor cell, positive regulation of angiogenesis and response to wounding (**Supplementary Figure S3** and **Supplementary Table S3**). Of the biological processes associated with genes showing downregulation, positive regulation of angiogenesis was particularly interesting given the observed link between angiogenesis and SS (53, 54).

The generation of RNA-seq datasets allowed us to also focus on broader changes in gene expression patterns that may be related to salivary gland dysfunction. We reasoned this approach would provide additional relevant insight into the pathophysiological features associated with this disease. Indeed, our analysis uncovered alterations to a number of genes (**Supplementary Figure S4**) that are associated with exocytosis in the acinar cells, a key biological process that can affect the secretion of salivary proteins and alter the contents of saliva. These results were noteworthy given the fact that one of the hallmark features of pSS is reduced salivary flow, which has been also documented in the NOD.B10 mouse model (49). Interestingly, we observed downregulation of a number of genes belonging to the Ras superfamily of GTPases (RAB GTPases). RAB GTPases play critical roles in exocytosis and membrane trafficking including vesicle formation, docking and membrane fusion, all vital functions necessary for proper saliva secretion (55). More specifically, we observed reduced gene expression levels of *Rabac1* and *Rab6b* in the salivary glands of SS mice compared to control animals (**Supplementary Figure S4**). Taken together, the transcriptomic profiling of the NOD.B10 mouse SMG has led to a better understanding of gene regulatory networks that are likely to be relevant for pSS pathobiology.

### Comparative Analysis of Primary Sjögren’s Syndrome in Mouse and Humans

**Figure 2:**
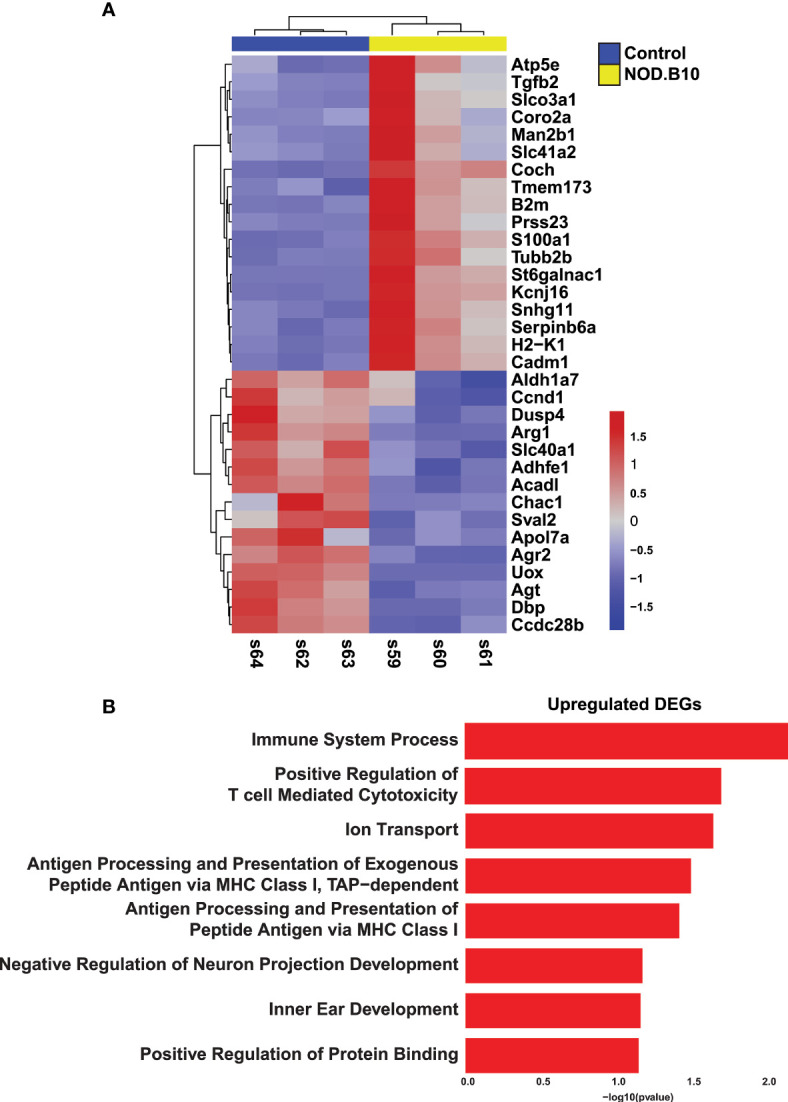
Comparative Analysis of Sjögren’s Syndrome Mouse Models. **(A)** Heatmap visualization of the common 33 overlapping genes identified in the RNA-seq datasets described here (Horeth et al.) and the Nguyen et al. (56) microarray dataset. **(B)** Bar plot highlights the biological processes enriched in the common 18 upregulated genes identified in panel **(A)** above.

In order to facilitate the discovery of genes and networks that may be important drivers of SS etiology, we next compared our results to microarray-based transcriptomic datasets generated from the well-established C57BL/6.NOD-*Aec1Aec2* mouse model of pSS (56). Comparison of the datasets revealed 33 common genes of which 18 were upregulated and 15 downregulated (**Figure 2A** and **Supplementary Table S4**). Despite the paucity of common genes between these two datasets, it is important to note that a number of these genes have been previously implicated in SS pathogenesis including *Prss23*, *Tmem173*, *Tgfb2, Dusp4*, and *Arg1* (57–61). To better appreciate the biological significance of the common genes, we performed pathway analysis on the 18 upregulated genes. As expected, we observed specific enrichment of biological processes associated with immune function including immune system processing, positive regulation of T cell mediated cytotoxicity, and general programs common to antigen processing and presentation (**Figure 2B** and **Supplementary Table S5**).

**Figure 3:**
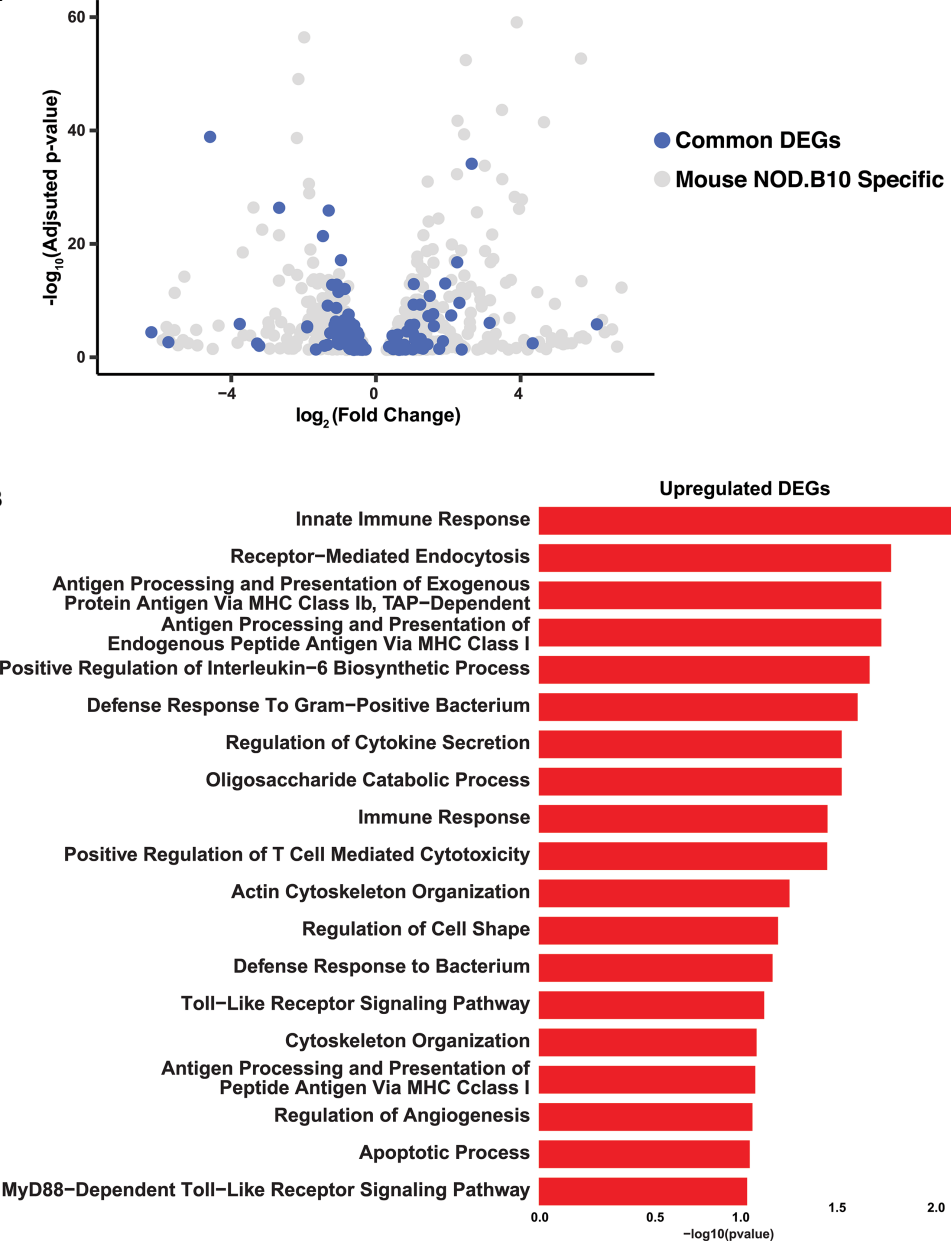
Integrated Analysis of Mouse and Human Sjögren’s Syndrome Datasets. **(A)** Volcano plot shows the distribution of statistically significant upregulated and downregulated DEGs in NOD.B10 SMGs (grey dots) and common DEGs (blue dots) between mouse and human SS. **(B)** Bar plot highlights the biological processes enriched in the common upregulated DEGs identified between the NOD.B10 and human pSS datasets.

Having established the transcriptomic repertoire of the NOD.B10 salivary glands, we wondered if there existed any commonalities between the mouse pSS salivary glands and those of human pSS patients. Towards this end, we compared the control and NOD.B10 salivary gland global transcriptome to RNA-seq datasets of human minor salivary glands of non-SS and pSS patients, which were recently generated in our lab (62). Of the 1076 DEGs identified in the NOD.B10 SMGs, we found 166 genes which showed concordant gene expression patterns to be shared across the two datasets. Of these DEGs, 57 were upregulated in both mouse and human while 109 were downregulated (**Figure 3A** and **Supplementary Table S6**). Notably, a number of common genes identified across both datasets have been previously demonstrated to play important roles in pSS pathogenesis including *Cxcl13*, *Tmsb10*, *Tap1*, *C1qa*, *Serpinb9*, and *Cds1* (14, 63–67) (**Figure 3A** and **Supplementary Table S6**). As expected, pathway analysis of the common upregulated genes revealed specific enrichment in biological processes important for innate immune response, antigen processing and presentation, regulation of cytokine secretion, toll-like receptor signaling, and Myd88-dependent toll-like receptor signaling pathway (**Figure 3B** and **Supplementary Table S7**). Conversely, shared downregulated genes showed enrichment in processes associated with O-glycan processing, ER to Golgi vesicle-mediated transport, carbohydrate metabolic process, and protein N-linked glycosylation *via* asparagine (**Supplementary Figure S5** and **Supplementary Table S8**). Interestingly, the observed downregulation of genes important for normal glycan function is in good agreement with the suggested role glycans play in influencing salivary flow, which is commonly reduced in pSS patients (68).

To further validate our findings, we next performed quantitative reverse-transcription polymerase chain reaction (qRT-PCR) using an independent set of salivary glands from control and NOD.B10 mice that were not included in our RNA-seq analysis. We focused on a select number of candidate genes that were common across both mouse and human datasets, several of which have been implicated in SS pathobiology. Consistent with our RNA-seq results, we found elevated mRNA expression levels of *Apoe*, *B2m*, *Tlr1*, *Def6*, *Entpd1*, and reduced expression levels of *Xbp1* (69–74) (**Supplementary Figure S6**).

### Single-Cell Characterization of NOD.B10 Salivary Gland Tissue

To follow up our observations from bulk RNA-seq experiments and to obtain a more detailed view of the cellular identities and states associated with SS pathology, we performed single-cell RNA-sequencing (scRNA-seq) analysis of adult control and NOD.B10 salivary glands. SMGs were isolated, dissociated into single cell suspensions and subjected to scRNA-seq using the 10X Genomics sequencing platform for single-cell capture to achieve in-depth expression profiling of individual SMG cells. After standard data processing and quality control procedures (see *Material and Methods*), we obtained transcriptomic profiles for 21,386 control cells and 13,846 NOD.B10 cells and performed downstream analysis (**Supplementary Figure S7**).

**Figure 4:**
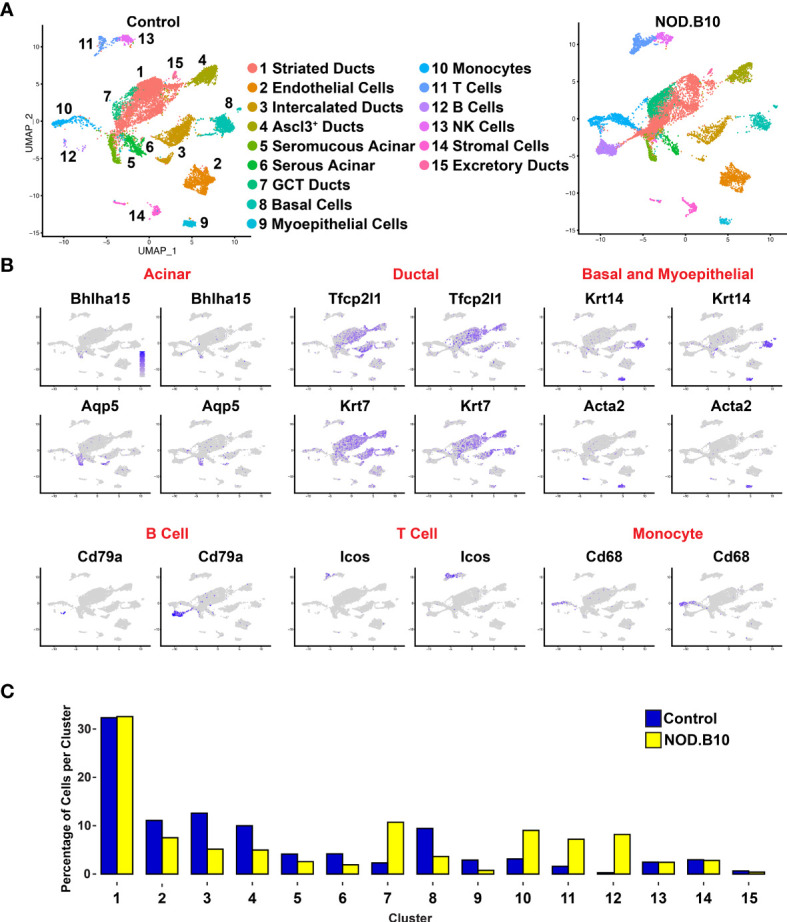
Single-cell RNA-sequencing Reveals Alterations in Cellular Heterogeneity in Mouse pSS SMGs. **(A)** Uniform manifold approximation and projection (UMAP) of control and NOD.B10 mouse SMGs. Cell cluster identities are also shown. **(B)** Feature plots demonstrate the expression of known acinar, ductal, basal and myoepithelial and immune genes. **(C)** Bar graph shows the proportion of cells per cluster.

**Figure 5:**
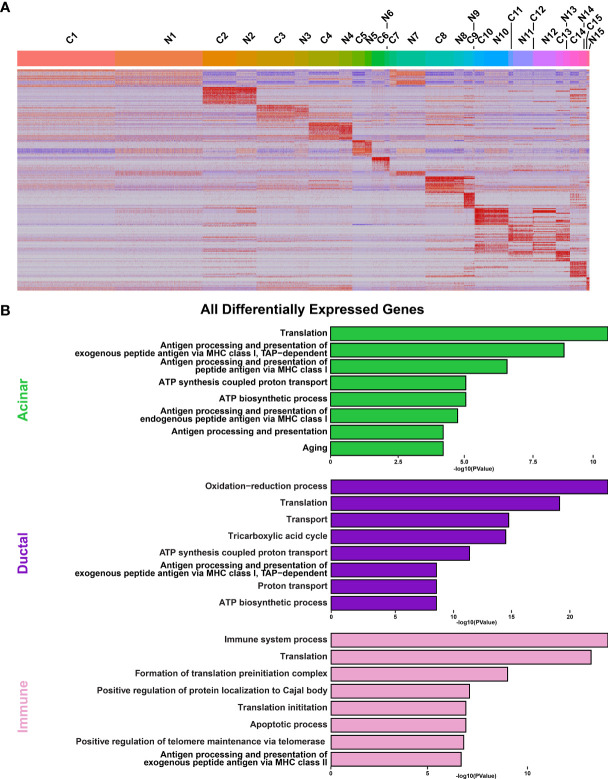
Analysis of Cellular Populations as Identified by Single-cell RNA-sequencing. **(A)** Heatmap visualization of the top 20 differentially expressed genes in each cluster as compared with all clusters between control (C) and NOD.B10 (N) SMGs. Upper bars represent cluster assignments. **(B)** DAVID analysis of all DEGs in the acinar (green), ductal (purple), and immune (pink) cell clusters.

In order to appreciate the level of cellular heterogeneity in the control glands, we first performed unsupervised clustering with affinity propagation based on the expression of high-variance genes. This analysis identified 15 clusters (C) of distinct cellular populations which we visualized *via* uniform manifold approximation and projection (UMAP) (**Figure 4A**). Cell type assignments were made based on the expression of known/validated marker genes, similar to what we and others have reported in the SGs (**Supplementary Tables S9**, **S10**) (43, 45, 47, 62, 75). A dot plot showing the expression of markers used for annotation is shown in **Supplementary Figure 8**. Interestingly, comparative analysis revealed striking differences between the various cell populations of the control and NOD.B10 samples (**Figure 4A**). These differences are further highlighted in **Figure 5A** in which the top 20 DEGs across all clusters between the control and NOB.B10 mice are shown (**Figure 5A** and **Supplementary Table S11**). As expected, we observed a dramatic increase in the number of immune cells in the NOD.B10 mouse salivary gland compared to control glands (**Figure 4**). This included elevated numbers of B cells, T cells, and myeloid cells as demonstrated by elevated mRNA expression levels of the cell type specific markers *Cd79a*, *Icos*, and *Cd68*, respectively (**Figure 4B**). This observation was further validated by examining the percentage of cells per cluster between control and NOD.B10 mice SMGs (**Figure 4C**). In stark contrast to the control SMG, the NOD.B10 sample consisted of reduced numbers of cells that comprise the epithelial cell populations with the NOD.B10 mice having a total of 3,626 epithelial cells (or ~34%) compared to 4,955 epithelial cells (or ~41%) observed in the control glands (**Figure 4**). This finding is not surprising given that the loss of these cell populations is commonly observed in pSS and has been attributed to the loss of salivary flow in patients (76). Overall, our scRNA-seq analyses has revealed the degree of cellular heterogeneity and the broad range of cell types associated with pSS diseased state.

**Figure 6:**
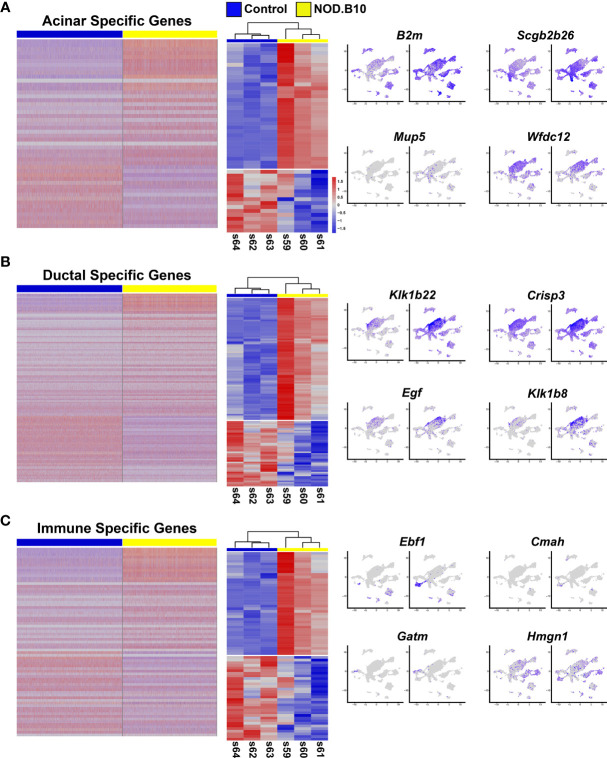
Comparative Analysis of the Bulk RNA-seq and Single Cell RNA-seq Datasets. **(A)** Heatmap visualization of DEGs identified in scRNA-seq datasets (left panel) which are common with the bulk RNA-seq datasets (middle panel) with representative feature plots (right panel) of genes in the acinar, ductal **(B)**, and immune cell clusters **(C)**.

To obtain a more detailed view of the molecular characteristics of the various cell populations we performed DEG analysis and concentrated on the 3 major cell types including acinar, ductal and immune cells from the control and NOD.B10 glands. Interrogation of the 2 acinar clusters (C5 and C6) of the control and NOD.B10 mice identified 388 DEGs with 263 genes showing upregulation and 125 showing downregulation (**Figure 6A** and **Supplementary Table S12**). We next utilized DAVID and Ingenuity Pathway Analysis (IPA) to explore the gene regulatory networks and pathways represented by the 388 DEGs. This analysis revealed enrichment of biological processes associated with translation and protein folding, which is not surprising given the secretory function of acinar cells (**Figure 5B** and **Supplementary Table S13**). Remarkably, enrichment of genes associated with immune cell processes including antigen processing and presentation, positive regulation of T cell mediated cytotoxicity and T cell receptor signaling were enriched in acinar cells of NOD.B10 glands (**Figure 5B** and **Supplementary Figure S9**).

We next performed similar DEG analyses focusing on the 5 ductal-specific clusters (C1, C3, C4, C7 and C15). This resulted in a total of 987 DEGs of which 543 were upregulated and 444 downregulated (**Figure 6B** and **Supplementary Table S14**). Interestingly, our pathway analyses of the 987 DEGs identified enrichment of pathways associated with immune cell function including antigen presentation and Eif2 signaling, the latter of which has been implicated in SS pathogenesis (77) (**Figure 5B**, **Supplementary Figure S10**, and **Supplementary Table S15**). Taken together these findings suggest that the acinar and ductal epithelial cells of the affected NOD.B10 glands may transition into a hybrid epithelial/immune cell-like state by aberrantly expressing molecules conventionally associated with immune cells. Finally, our DEG analysis of the 4 immune cell clusters (C10, C11, C12, C14) identified 1215 DEGs, with 793 being upregulated and 422 genes downregulated (**Figure 6C** and **Supplementary Table S16**). As expected, our pathway analyses of the 1215 DEGs revealed enrichment of processes associated with immune specific functions including immune system process and response as well as antigen presentation (**Figure 5B**, **Supplementary Figure S11** and **Supplementary Table S17**).

Given that the bulk RNA-seq and scRNA-seq were independent experiments performed on separate control and NOD.B10 SMG samples, we next sought to compare these datasets. For this purpose, we leveraged the scRNA-seq derived acinar, ductal, and immune cell cluster gene signatures and performed comparative analyses with the bulk RNA-seq dataset. Comparison of the 388 acinar DEGs with the 1076 DEGs obtained from the bulk RNA-seq studies identified 48 genes which showed concordant gene expression patterns with 32 showing upregulation and 16 being downregulated (**Figure 6A** middle panel and **Supplementary Table S18**). Interestingly, while a number of the upregulated genes showed widespread expression based on feature plot analysis (**Figure 6A** right panel), we did identify beta-2-microglobulin (*B2m*) which has been shown to be associated with increased disease severity in patients with pSS (2, 70). Among the 987 ductal DEGs, we found 129 genes that showed concordant gene expression patterns with 84 being upregulated and 45 downregulated in the bulk RNA-seq data (**Figure 6B** middle panel and **Supplementary Table S19**). Of note, several of these genes belong to the kallikrein family of serine proteases, which have recently been linked to pSS as well as other autoimmune diseases (**Figure 6B** right panel) (78–80). Finally, similar analyses of the immune cell clusters revealed that of the 1215 DEGs detected between the control and NOD.B10 scRNA-seq datasets, 71 genes showed concordant gene expression patterns with 39 being upregulated and 32 showing downregulation (**Figure 6C** middle panel and **Supplementary Table 20**). Of these genes, we found early B cell factor 1 (*Ebf1*) and high mobility group nucleosome binding domain 1 (*Hmgn1*) to be upregulated (**Figure 6C** right panel). While *Ebf1* and *Hmgn1* have not been directly linked to SS, they have been shown to play important roles in immune function and may serve as potential candidates for future follow-up studies (81–83). While the DEGs common between the bulk RNA-seq and scRNA-seq datasets might seem relatively modest at first glance, we suspect that this might be in part due to poor gene coverage (from expressional dropout for instance) for the scRNA-seq data. Nevertheless, the comparative analysis does provide a reasonable number of genes with high confidence that are likely to be critical to the underlying pSS pathogenic state of the NOD.B10 SMGs.

### Intrinsic Activation of Salivary Gland Epithelial Cells Contribute to Immune Dysregulation

**Figure 7:**
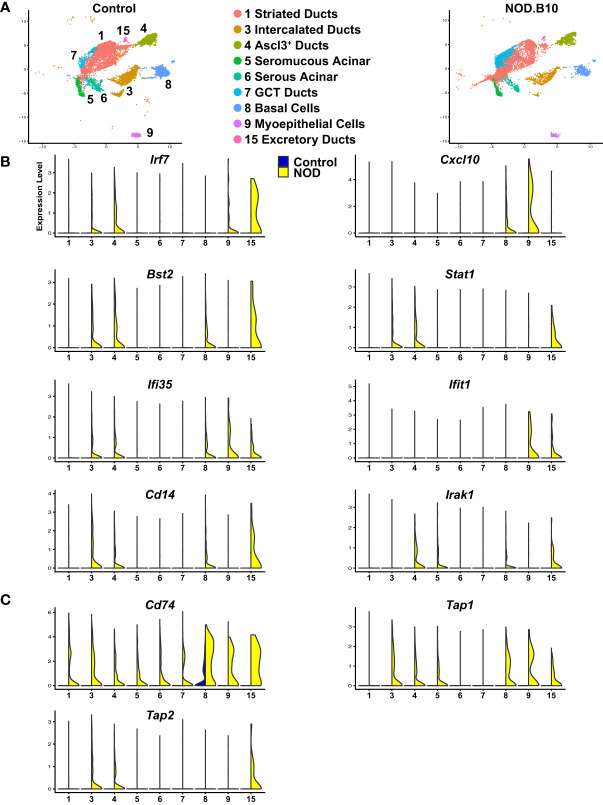
Activation of Immune Responses of Mouse SS Salivary Gland Epithelial Cells. **(A)** UMAP visualization of the epithelial cell populations of control and NOD.B10 mouse SMGs based on hierarchical clustering analysis performed in **Figure 4A**. Epithelial cell cluster identities are shown. **(B, C)** Violin plots demonstrate expression of various immune related genes in the epithelial cell populations of control and NOD.B10 mouse SMGs.

>While salivary gland dysfunction has been commonly thought to occur as a consequence of abnormal B cell and T cell responses, emerging evidence suggests a functional role for the epithelial cells in contributing to disease development and progression. Indeed, salivary gland epithelial cells isolated from patients with SS have been shown to play an active role in driving the initial local autoimmune responses by mediating recruitment, homing, activation, and differentiation of immune cells (34, 35). To better appreciate the functional role of the epithelial cells, we mined our scRNA-seq datasets from control and NOD.B10 mice and focused on the 9 epithelial cell clusters and performed differential gene expression analysis (**Figure 7A** and **Supplementary Table S21**). This analysis identified a number of upregulated DEGs in the epithelial cells of the NOD.B10 glands that have been shown to play important roles in immune cell responses. IFN signaling has emerged as a key driver in the pathogenesis of pSS with patients demonstrating elevated expression levels of genes associated with this signaling pathway (84, 85). In agreement with these findings, our analysis revealed a number of molecular players involved in IFN signaling to be markedly upregulated in the epithelial cells of the NOD.B10 mice compared to control cells. For instance, we identified interferon-regulatory factor 7 (*Irf7*), chemokine (C-X-C motif) ligand 10 (*Cxcl10*), bone marrow stromal cell antigen 2 (*Bst-2*), signal transducer and activator of transcription 1 (*Stat1)*, interferon induced irotein 35 (*Ifi35)* and interferon-induced protein with tetratricopeptide repeats 1 (*Ifit1)* to be enriched in the epithelial cell clusters of the NOD.B10 mice (**Figure 7B**) (86).

In addition to genes involved in IFN signaling, we observed upregulation of *Cd14*, a GPI-anchored pattern recognition receptor that functions in innate immune responses (87). More specifically, Cd14 is a co-receptor in the toll like receptor 4 (TLR4) complex, activation of which results in the recruitment of Myd88 and various IL-1R associated kinases (IRAKs) leading to NF-κB and MAPK signaling pathway activation (88, 89). Not surprisingly, we also observed elevated gene expression levels of *Irak1*, an important mediator of this signaling cascade (90) (**Figure 7B**). In line with these findings, several genes involved in antigen processing and presentation were also selectively upregulated in the NOD.B10 salivary epithelial cells including *Cd74*, transporter associated with antigen processing 1 (*Tap1*), and *Tap2* (**Figure 7C**) (91, 92). Taken together, our results suggest that similar to immune cells, epithelial cells appear to play a reactive role in driving the immune related changes associated with pSS.

## Discussion

Primary Sjögren’s Syndrome is a complex systemic autoimmune disease with no known cause or effective cure. Despite extensive research efforts over the last several decades, the underlying molecular mechanisms driving SS pathogenesis, remains elusive. Here we have performed a comprehensive transcriptomic characterization of the underlying global circuitry in the salivary glands of a well-established pSS mouse model. In parallel, we have utilized single-cell RNA-seq to interrogate alterations in the cellular heterogeneity of mouse pSS salivary glands and have described the immuno-reactive state of the epithelial cells suggesting that they are active participants in pSS pathogenesis. Our multipronged genomic and genetic approach has revealed novel insight into the transcriptional regulatory circuitry and the various cell types spurring the immune related changes underlying this disease.

Mouse models have long served as valuable model systems to study various aspects of human disease. Over the years, SS mouse models have provided a myriad of information regarding different aspects of SS pathogenesis including onset, progression, and treatment. While these studies have been invaluable in providing insight into the complex nature of this disease, no studies to date have comprehensively dissected the cellular heterogeneity and examined the various cellular states of the salivary glands of pSS mice. Although it is no surprise that our scRNA-seq analysis identified increased populations of immune cells in the glands of pSS mice, our findings uncovering the degree to which the epithelial cells exist in an immune activated state and expressed markers that are commonly associated with immune cell function, was striking. It is tempting to speculate that the NOD.B10 epithelial ductal cells are transitioning and displaying a similar gene expression profile to that of immune cells. However, this requires further investigation. While the contribution of pSS epithelial cells has received some attention (33–35), future studies aimed at investigating the transcriptional changes the epithelial cells undergo as they transition to this activated state will be important. Moreover, identifying the signals that drive the epithelial activated state will be critical to understanding how they contribute to this disease.

Our comparative transcriptomic analysis of mouse and human pSS glands allowed us to identify genes that are common and potentially relevant in driving the underlying molecular mechanisms contributing to this disease. Interestingly, our analysis revealed that of the 1076 DEGs we identified in the SGs of the NOD.B10 mice, only 166 genes were common to the human RNA-seq studies of pSS and non-pSS salivary gland tissue (62). While this number was surprisingly low, the discrepancies can be attributed to a number of factors including the inherent differences between the types of glands included in our analysis. For example, in the current study we compared NOD.B10 submandibular salivary glands datasets to human minor salivary glands. It is also plausible that the modest number of overlapping genes we observed may be due to differences in disease stages or a reflection of the complex nature of this disease. Despite the modest overlap, in addition to identifying conserved networks and pathways, our analysis also identified a number of common genes which have been previously reported to play key roles in SS. In addition, we uncovered several genes that have not been previously associated with this disease and which may serve as candidates for future studies. For instance, we identified a number of genes involved in sialic acid biosynthesis and sialylation including glucosamine (UDP-N-acetyl)-2-epimerase/N-acetylmannosamine kinase (*Gne*), an important enzyme in the sialic acid biosynthetic pathway. Although *Gne* has not been directly linked to SS, sialic acids have been shown to play important roles in autoimmunity (93). Additionally, we identified cytidine monophosphate N-acetylneuraminic acid synthetase (*Cmas*), a catalyzing enzyme involved in the sialylation pathway, to be a common gene between mouse and human SS. While no direct link to SS has been reported, *Cmas* has been demonstrated to direct various immune responses (94, 95).

The wealth of transcriptomic data from the mouse model of pSS described in this paper is a critical first step in understanding both the cellular and molecular mechanisms of this disease. One major advantage of the NOD.B10 model is the possibility of performing a time course of genomic studies that addresses the nuances of the gradual and progressive nature of pSS pathology. Indeed, this approach will likely uncover the early triggers and precipitating factors that contribute to the etiology of pSS. Future studies, particularly those that take advantage of single cell techniques such as spatial transcriptomics, are also needed to reveal the topological map of the niche in which subtypes of various epithelial and immune cells co-exist, crosstalk with each other and more importantly, contribute to SMG dysfunction in pSS.

## Data Availability Statement

The datasets presented in this study can be found in online repositories. The names of the repository/repositories and accession number(s) can be found below: https://www.ncbi.nlm.nih.gov/geo/, GSE175649.

## Ethics Statement

The animal study was reviewed and approved by State University of New York at Buffalo (University at Buffalo) Institutional Animal Care and Use Committee (IACUC).

## Author Contributions

EH, AO, E-ACS, MC, JB, SM, JK, JMK, SS, and R-AR all contributed to performing experiments, data acquisition, analysis, and interpretation. R-AR contributed to conception, design and drafted the manuscript. EH, AO, E-ACS, MC, JB, SM, JK, JMK, and SS critically revised the manuscript. All authors contributed to the article and approved the submitted version.

## Funding

This work was supported by National Institutes of Health/National Institute of Dental and Craniofacial Research (NIH/NIDCR) training grant (NIH/NIDCR) DE023526 to the State University of New York at Buffalo, School of Dental Medicine, Department of Oral Biology which supported EH, E-ACS, MC, and SM.

## Conflict of Interest

The authors declare that the research was conducted in the absence of any commercial or financial relationships that could be construed as a potential conflict of interest.

## Publisher’s Note

All claims expressed in this article are solely those of the authors and do not necessarily represent those of their affiliated organizations, or those of the publisher, the editors and the reviewers. Any product that may be evaluated in this article, or claim that may be made by its manufacturer, is not guaranteed or endorsed by the publisher.
