# Supplementary material for: Transcriptomic and Single-Cell Analysis Reveals Regulatory Networks and Cellular Heterogeneity in Mouse Primary Sjögren’s Syndrome Salivary Glands
Source: Front Immunol. 2021 Nov 29;12:729040. doi: 10.3389/fimmu.2021.729040 (PMC8666453; doi:10.3389/fimmu.2021.729040)
Supplement: Supplementary file 1 [file DataSheet_1.docx]

**Supplementary Figure S1. Histological Examination of Control and NOD.B10 SMGs.** H&E staining of control and NOD.B10 SMGs from female mice. Compared to control glands, NOD.B10 mice show areas of immune cell infiltration (black arrows). Scale bar 37μm.


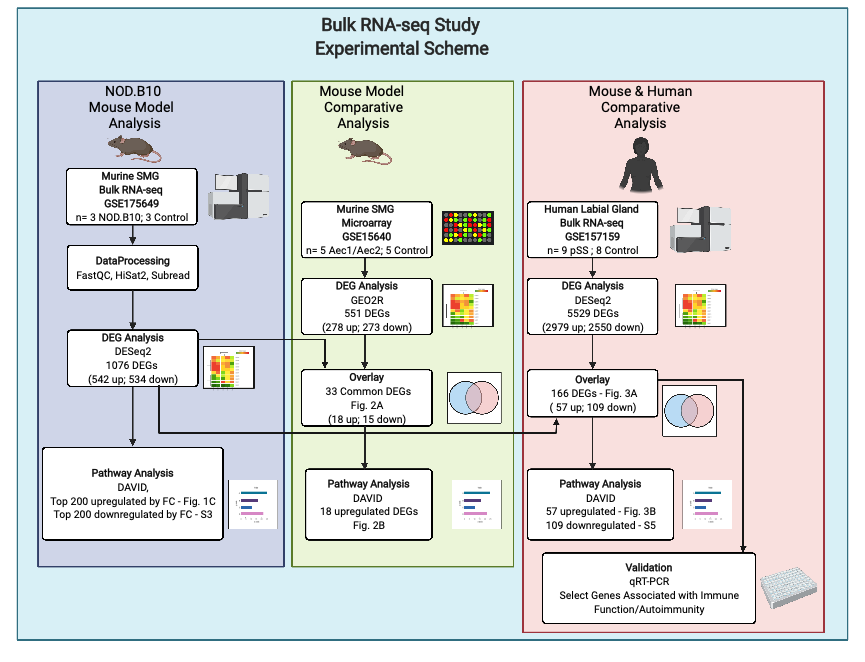


**Supplementary Figure S2.** Overview of RNA-sequencing data processing and analysis steps.

**Supplementary Figure S3. Enriched Biological Processes of Downregulated Genes.** Bar plot highlights the biological processes enriched in the downregulated DEGs identified between the control and NOD.B10 SMGs.

**Supplementary Figure S4. Changes in Exocytosis in NOD.B10 SMGs.** Heatmap visualization of select DEGs in NOD.B10 SMGs that have been shown to function in exocytosis.

**Supplementary Figure S5. Enriched Biological Processes of Common Downregulated Genes.** Bar plot highlighting enriched biological processes in the common downregulated DEGs identified between the mouse and human SS datasets.

**Supplementary Figure S6.** **Validation of Select Genes**. Quantitative RT-PCR analysis validating the mRNA expression levels of a panel of select genes in control and NOD.B10 mouse SMGs that were not subjected to RNA-seq analysis. Values were normalized to the housekeeping gene HPRT. Data are represented as ± S.D (n=3). *p<0.05, **p<0.01, ***p<0.001

**Supplementary Figure S7.** Overview of scRNA-sequencing data processing and analyses steps.

**Supplementary Figure S8.** Dot plot showing expression of markers used for annotation of SMG for scRNA-seq experiments.


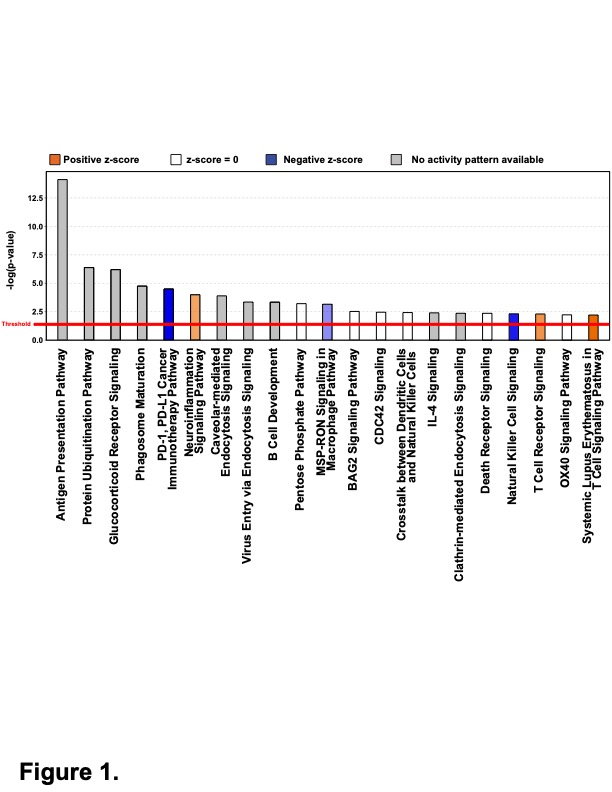


**Supplementary Figure S9.** Top 21 significantly affected canonical pathways for Acinar specific DEGs identified in our scRNA-seq dataset based on IPA.


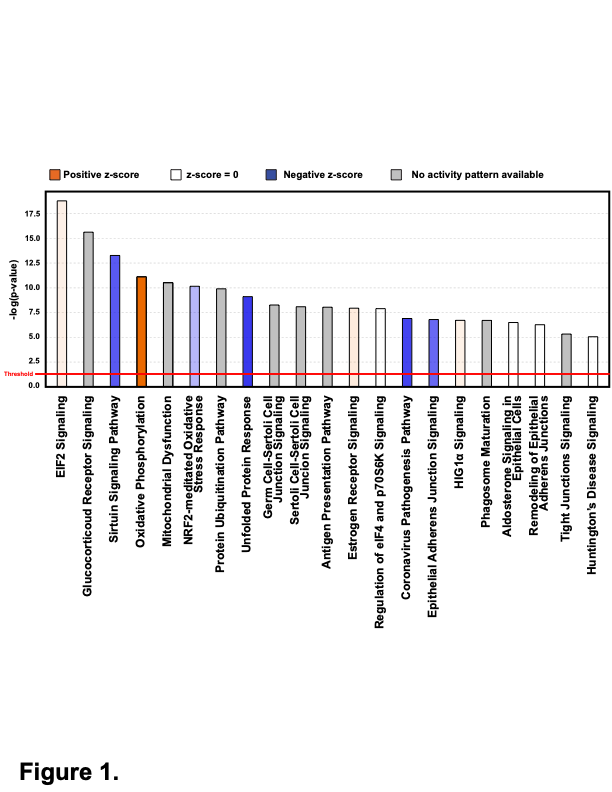


**Supplementary Figure S10.** Top 21 significantly affected canonical pathways for Ductal specific DEGs identified in our scRNA-seq dataset based on IPA.


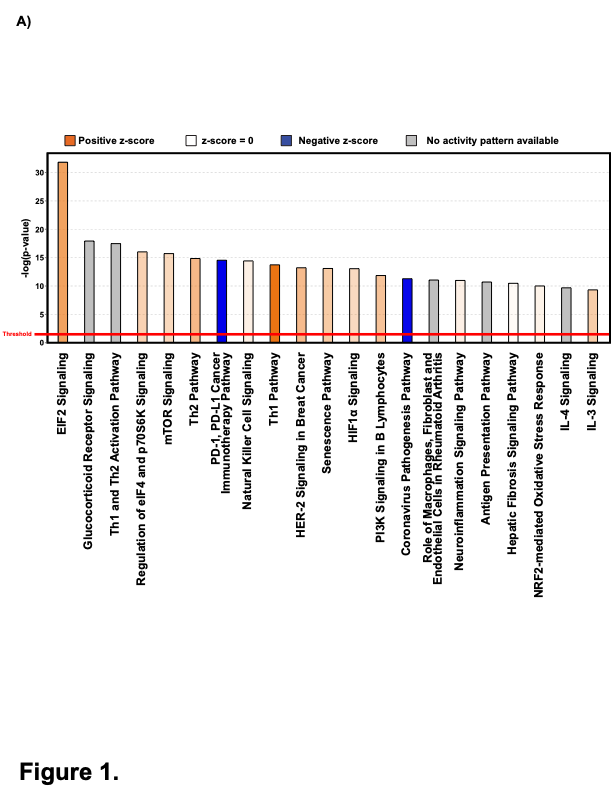


**Supplementary Figure S11.** Top 21 significantly affected canonical pathways for Immune specific DEGs identified in our scRNA-seq dataset based on IPA.
